# Succinate Dehydrogenase Loss in Familial Paraganglioma: Biochemistry, Genetics, and Epigenetics

**DOI:** 10.1155/2015/296167

**Published:** 2015-07-29

**Authors:** Yeng F. Her, L. James Maher

**Affiliations:** ^1^Department of Biochemistry and Molecular Biology, Mayo Clinic College of Medicine, 200 First Street SW, Rochester, MN 55905, USA; ^2^Mayo Clinic Medical Scientist Training Program, Mayo Clinic College of Medicine, 200 First Street SW, Rochester, MN 55905, USA

## Abstract

It is counterintuitive that metabolic defects reducing ATP production can cause, rather than protect from, cancer. Yet this is precisely the case for familial paraganglioma, a form of neuroendocrine malignancy caused by loss of succinate dehydrogenase in the tricarboxylic acid cycle. Here we review biochemical, genetic, and epigenetic considerations in succinate dehydrogenase loss and present leading models and mysteries associated with this fascinating and important tumor.

## 1. Introduction: Overview of Glucose Metabolism

**Figure 1:**
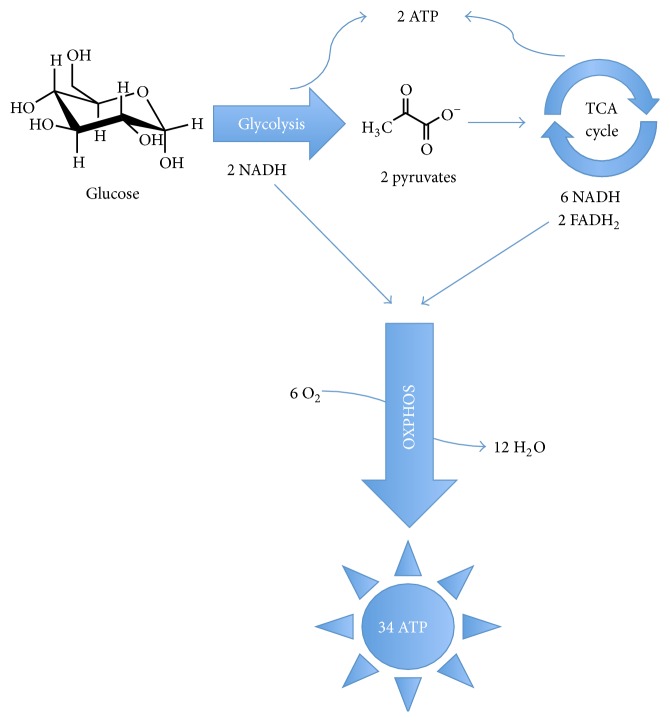
Overview of ATP production in eukaryotic cells. Complete oxidation of glucose to water and carbon dioxide occurs in three processes: glycolysis, the TCA cycle, and oxidative phosphorylation (OXPHOS).

Glucose metabolism is a central source of energy [1]. Glucose also provides metabolic intermediates for biosynthesis of nucleotides, fatty acids, amino acids, and coenzymes. Glucose metabolism to produce carbon skeletons and ATP occurs in three stages: glycolysis, the TCA (tricarboxylic acid) cycle, and oxidative phosphorylation (OXPHOS) (Figure 1). Glycolysis occurs in ten steps in the cytoplasm of cells, producing from each glucose molecule a net of two ATP molecules and two NADH molecules (Figure 1). Under aerobic conditions, NADH produced from glycolysis is oxidized to NAD+ in the electron transport chain (ETC). The resulting high-energy electrons are transferred to oxygen yielding water and additional molecules of ATP by oxidative phosphorylation driven by a proton gradient formed in mitochondria by the electron transfer process. In contrast, under anaerobic conditions, NADH cannot be reoxidized to NAD+. Since NAD+ is required for glycolysis to continue, NADH reduces pyruvate to lactate, resulting in regeneration of NAD+.

Under aerobic conditions, pyruvate is oxidized by the pyruvate dehydrogenase complex, located in the mitochondria, to the acetyl function of acetyl-coenzyme A (acetyl-CoA) [1]. The TCA cycle then has eight steps whereby citrate, formed from acetyl-CoA and oxaloacetate, is transformed into seven intermediate metabolites releasing two molecules of carbon dioxide and one molecule of each of NADH and FADH_2_. Per turn of the TCA cycle, there are four oxidation steps where energy is released and stored in the form of three NADH molecules and one FADH_2_ molecule (Figure 1). One GTP is also produced when succinyl-CoA is converted to succinate.

**Figure 2:**
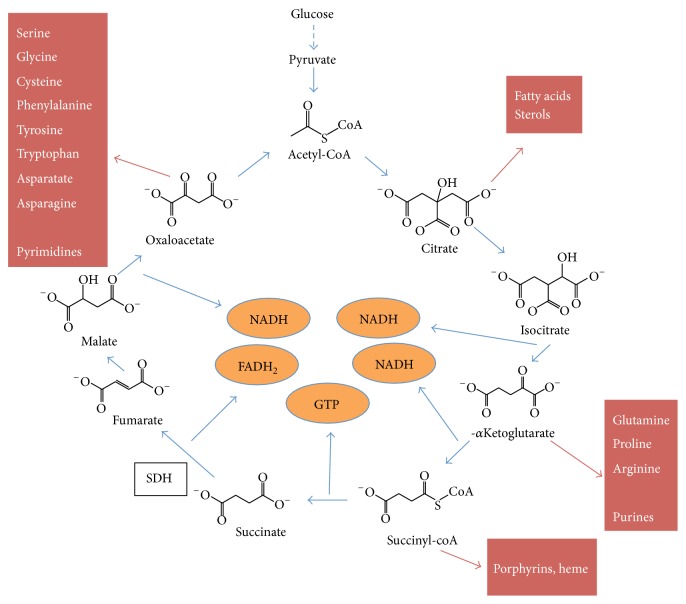
TCA cycle metabolites in anabolism. TCA cycle metabolites are precursors in a wide spectrum of biosynthetic pathways. In the boxes are final products of anabolism from the four TCA cycle metabolites indicated by red arrows.

The TCA cycle is not simply a path for generating ATP by glucose oxidation. The cycle also generates metabolites for many biosynthetic pathways (Figure 2) [1]. Thus, anaplerotic pathways exist to replenish metabolites extracted from the TCA cycle. Examples include production of oxaloacetate from pyruvate or aspartate, production of *α*-ketoglutarate (*α*-KG) from glutamate, production of succinyl-CoA from fatty acids, and production of fumarate from adenylosuccinate.

The final stage in glucose metabolism is oxidative phosphorylation. This process depends upon five enzyme complexes (Complex I through Complex V) embedded in the inner mitochondrial membrane. Complexes I, III, and IV are proton pumps that generate an electrochemical gradient across the inner mitochondrial membrane, storing the REDOX energy associated with the high-energy electrons extracted from glucose [1]. Complex V then uses this proton-motive force to synthesize ATP. Electrons enter this path from the TCA cycle. Succinate is oxidized to fumarate by succinate dehydrogenase (Complex II). Protons flow passively from the intermembrane space (high concentration) to the matrix (low concentration) through a proton pore in the *F*_*o*_ subunit of Complex V (ATP synthase) converting electrochemical gradient energy to protein dynamics required for ATP synthesis catalyzed by the *F*_1_ subunit on Complex V. The overall energy gain from oxidative phosphorylation is 34 ATP per one molecule of glucose (Figure 1).

## 2. Metabolism and Cancer

Almost a century ago, Warburg made the observation that the metabolism of cancer cells differs from normal cells. Lactate production in slices of normal tissue was only observed upon oxygen deprivation [2]. Remarkably, tumor tissue slices produced large amounts of lactate even when oxygen was available [3]. This led to the hypothesis that cancer cells prefer glycolysis for ATP production even in the presence of oxygen. This is in stark contrast to the observation that normal cells produce ATP from oxidative metabolism of glucose utilizing glycolysis, the TCA cycle, and oxidative phosphorylation.

Energy produced through glycolysis yields two molecules of ATP per one molecule of glucose. This is far less than the 34 molecules of ATP produced per molecule of glucose through oxidative phosphorylation [1]. Why might cancer cells prefer this very poor ATP yield, apparently wasting most of the available energy in the form of partially oxidized carbon skeletons? It remains unclear how aerobic glycolysis causes, correlates with, or facilitates cancer progression. Three hypotheses have been proposed. First, the hypoxic tumor microenvironment selects for cells that have switched to a constitutive glycolytic metabolism. Second, aerobic glycolysis is better suited for providing carbon skeletons for cell proliferation. Third, cancer cells induce a Warburg-like metabolic reprogramming of the microenvironment and exploit these by-products for growth. All three hypotheses imply that efficient energy extraction from glucose is not important.

Tumor vasculature is disorganized, immature, tortuous, and hyperpermeable [4]. Perfusion is ineffective and inefficient [5], imposing nutrient and hypoxic stress on the growing tumor [6–8]. This stress triggers the stabilization of hypoxia-inducible transcription factors (HIFs), which stimulate angiogenesis and increased expression of glycolytic enzymes, glucose transporters, and inhibitors of mitochondrial metabolism [9]. This selective pressure forces tumor cells to decrease their dependence on aerobic metabolism and shift to glycolysis. If this shift becomes irreversible, the Warburg observation that cancer cells produce large amounts of lactate even in room air is explained. Indeed, some cancer cells undergo aerobic glycolysis even upon high oxygen perfusion [10–12].

In contrast to the hypothesis that cancer cells that preferentially rely on glycolysis are selected by hypoxia, the biomass hypothesis is based on the idea that aerobic glycolysis is superior to the TCA cycle for providing necessary carbon skeletons required for cancer cell proliferation [13]. Thus, human physiology provides an abundance of blood glucose and not all glucose should be used to generate ATP [13]. Requirements for biosynthesis of nucleotides and amino acids are particularly important for cancer cell growth [14]. When cancer cells cease aerobic glycolysis upon inhibition of lactate dehydrogenase, tumor cell proliferation is compromised. This suggests that aerobic glycolysis is crucial for cancer growth [15].

Lastly, it is known that interplay with the tumor microenvironment can promote an epithelial-mesenchymal transition in cancer cells, which contributes to cancer progression and metastasis. This interaction is not exclusively one-sided as originally thought. It has been argued that cancer cells can reciprocate by inducing metabolic reprogramming of cancer-associated fibroblasts (CAFs) to increase glucose uptake and lactate production, while simultaneously increasing tumor lactate receptor expression [16]. This reciprocal conditioning of CAFs is proposed to allow cancer cells to exploit their microenvironment for metabolic advantage. Indeed, this has been observed in a neuroblastoma SK-N-AS* SDHB*-silenced cell culture model [17].

## 3. Succinate Dehydrogenase: Structure, Regulation, and Assembly

### 3.1. SDH Structure

This review focuses on the effects of loss of succinate dehydrogenase (SDH). SDH is part of both the TCA cycle and the ETC. In the TCA cycle, SDH catalyzes the oxidation of succinate to fumarate producing one molecule of FADH_2_. In the ETC, SDH is known as Complex II, the only complex encoded by nuclear DNA. Electrons from succinate oxidation are transferred to ubiquinone in the ETC [18].

**Figure 3:**
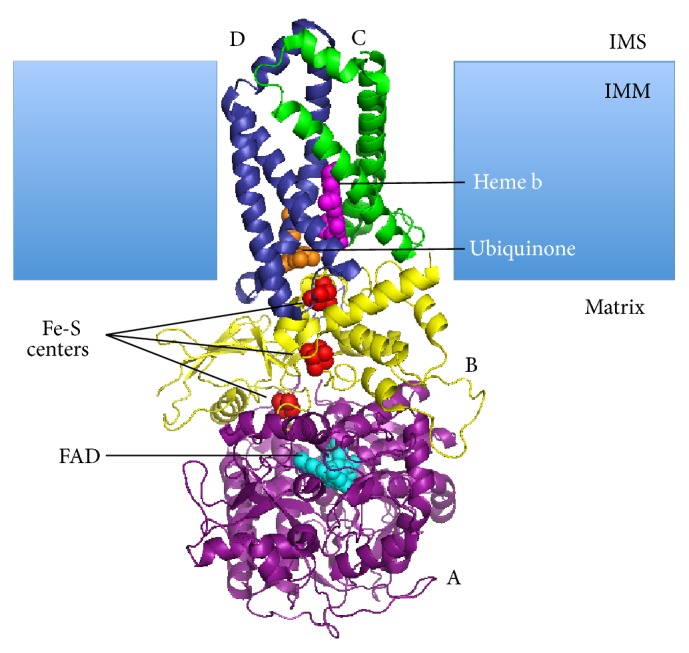
Structure of succinate dehydrogenase [20]. The enzyme is conserved through evolution and has two transmembrane subunits (SDHC and SDHD) and two matrix subunits (SDHA and SDHB). SDHA contains a bound FAD cofactor and the binding site for succinate. SDHB has three Fe-S centers. Heme b and ubiquinone are sandwiched between SDHC and SDHD. Electron pairs move from the substrate succinate to FAD, through the three Fe-S centers, and then to ubiquinone. Heme b prevents reactive oxygen species formation and does not participate in electron transfer. IMS (intermembrane space); IMM (inner mitochondrial membrane).

Structurally, the SDH complex has four subunits, SDHA, SDHB, SDHC, and SDHD (Figure 3). SDHC and SDHD contain hydrophobic components that anchor the complex to the inner mitochondrial membrane. Protruding into the matrix are SDHA and SDHB. SDHA is covalently attached to a flavin adenine dinucleotide (FAD) prosthetic group. SDHA carries the binding site for succinate. Upon succinate binding, SDHA brings succinate into juxtaposition with the isoalloxazine ring of FAD, where oxidation to fumarate is catalyzed [18]. SDHB connects SDHA to SDHC and SDHD. SDHB contains three Fe-S centers that mediate electron transfer from succinate to ubiquinone [19]. There are two ubiquinone-binding sites on the SDH complex [19, 20]. The high-affinity site is formed by residues from SDHB, SDHC, and SDHD [21, 22], located near the matrix side of the inner mitochondrial membrane. The low-affinity site is formed by SDHC and SDHD, located closer to the intermembrane space of the inner mitochondrial membrane. Lastly, a heme *b* group is sandwiched between SDHC and SDHD, presumably scavenging free electrons to prevent the formation of reactive oxygen species.

### 3.2. Regulation of SDH Activity

SDH activity can be modulated through succinate competitors, ubiquinone inhibitors, or posttranslational modifications. Succinate competitors include malonate, malate, and oxaloacetate [23, 24]. Structurally, these compounds are similar to succinate, which explains their ability to compete for SDH binding. Notably, both malate and oxaloacetate are TCA cycle metabolites. Carboxin and thenoyltrifluoroacetone are synthetic ubiquinone inhibitors that block electron transfer from the SDH complex to ubiquinone [25, 26].

In terms of posttranslational modifications, phosphorylation and acetylation of SDHA lysine residues have been shown to modulate SDH activity [27, 28]. For instance, knockout of SIRT3, a major deacetylase, resulted in accumulation of SDHA lysine acetylation. This decreased the activity of the SDH complex. Upon reexpression of SIRT3, SDHA lysine acetylation was removed and SDH activity was restored [28]. Similarly, phosphorylation of SDHA lysine residues has similar effects on SDH activity [27]. Succinylation of SDHA lysine residues has recently been reported in cells treated with an SDH inhibitor or upon knockdown of succinyl-CoA synthetase [29, 30]. Succinyllysine modification may modulate SDH activity.

### 3.3. Assembly of SDH

SDH is an intricate complex with four subunits each containing different components that must be properly assembled into the complete enzyme. This presents multiple coordination challenges. In response, protein chaperones facilitate protein folding and factor insertion. These proteins include products of the* tcm62*,* flx1*,* SDHAF1*,* sdh5/SDHAF2*,* SDHAF3*, and* sdh8/SDHAF4* genes.

Dibrov and colleagues were the first to identify Tcm62 in a genetic screen for mammalian mutations that induced loss of SDH activity [31]. The* tcm62* gene encodes a mitochondrial membrane protein that was shown to directly interact with three SDH subunits. Klanner and colleagues confirmed this hypothesis by showing that Tcm62 supported protein folding under heat stress and was required for mitochondrial gene expression [32]. The role of Tcm62 as a general protein folding chaperone was further supported by Heiden and colleagues [33].

During the Sdh1 (yeast ortholog of mammalian SDHA) assembly process, FAD must be imported from the cytosol to the mitochondrial matrix and inserted into the Sdh1 subunit. This is accomplished by Flx1 (flavin exchange) protein. Flx1 is located in the inner mitochondrial membrane and is a member of a superfamily of mitochondrial carriers that exchange substrates between the cytosol and the matrix [34]. The role of Flx1 as a FAD transporter was first described by Tzagoloff and colleagues [35]. They observed that* flx1* mutant strain had a decreased mitochondrial flavin adenine dinucleotide/flavin mononucleotide (FAD/FMN) ratio, suggesting that Flx1 is a mitochondrial FAD importer.

The role of Flx1 in FAD insertion into Sdh1 was described by Hao and colleagues [36]. These authors reported complete loss of covalent FAD incorporation into Sdh1 in* flx1* mutant cells and inability of the mutant to grow on nonfermentable carbon sources. The* flx1* mutant was also hypersensitive to hydrogen peroxide [37]. Interestingly, overexpression of Sdh5 (a protein required for flavination of SDH) partially restored SDH activity in the* flx1* mutant.

SDHAF protein family members can be divided into three groups: those that insert Fe-S centers into SDHB, those that insert FAD into SDHA, and those that participate as chaperones. SDHAF1 and SDHAF3 insert Fe-S centers [38–40]. SDHAF1 mutation was reported in an Italian family with progressive infantile leukoencephalopathy and decreased SDH activity [38]. Using a yeast model, a* sdhaf1* mutant showed significant loss of SDH activity and inability to grow on acetate as the carbon source. Its role in Fe-S insertion was brought to light by the discovery that Sdhaf1 and Sdhaf3 act together to mediate Fe-S cluster maturation in Sdh2 by protecting the protein from reactive oxygen species [40]. Sdhaf1 or Sdhaf3 deficient yeast and* Drosophila* specifically showed impaired Sdh2 protein with loss of SDH activity.

*Sdh5* and* Sdh8* gene products are both SDH assembly factors that facilitate FAD covalent interaction into sdh1. Hao and colleagues showed that mutant* sdh5* lacked SDH activity [35]. When Sdh5 was overexpressed, it rescued the FAD incorporation defect previously observed in* flx1* mutant cells, which led to the assertion that Sdh5 is required for FAD insertion into the catalytic Sdh1 subunit. This conclusion has been confirmed in multiple studies [41–43].

In a recent study using yeast,* Drosophila*, and mammalian cells, Sdh8 was shown to interact with Sdh1 in the mitochondrial matrix and mediated interaction with Sdh2 [44]. The authors concluded that Sdh8 interacts with flavo-Sdh1 in the matrix and stabilizes it before Sdh1-Sdh2 is assembled into the SDH complex.

## 4. Hereditary Paraganglioma

Paraganglioma (PGL) is a rare neuroendocrine neoplasm derived from neural crest cells located between the pelvic floor and the base of the skull. It has an annual incidence of approximately 0.8 per 100,000 [45]. PGL tumors can be derived from cells of the parasympathetic or sympathetic ganglia. These two PGL types occur with similar frequency but have distinct anatomical locations and clinical features [46]. PGLs arising from the parasympathetic ganglia are mainly located around the head and neck (carotid body, glomus jugulare, and glomus typanicum) [46], are typically benign, and are rarely associated with catecholamine secretion [47–49]. Sympathetic PGLs are located in the abdomen and often secrete catecholamines such as epinephrine, norepinephrine, and dopamine. PGL tumors found in the adrenal gland are called pheochromocytomas. Patients with sympathetic PGLs often develop episodic hypertension and are at a higher risk for malignancy [50–54]. Most PGLs are diagnosed between the third to fifth decades of life, with sympathetic PGLs discovered earlier compared to parasympathetic PGLs [55].

There is interesting evidence that PGL development may be associated with chronic hypoxia. The incidence of benign head and neck PGL (carotid body tumors) can approach 1 in 10 for high altitude inhabitants compared to 1 in 500,000 in low altitude dwellers [56–59]. In bovines, the incidence of PGL can be 50% at high altitude [59]. PGLs are also more common in patients with chronic obstructive lung disease and cyanotic congenital heart disease [60–62]. The reason for this association is still unknown and is a subject of this review. We propose here that increased PGL incidence and growth reflect inhibition of *α*-KG-dependent dioxygenases by hypoxia, an effect that we show to be synergistic with succinate accumulation when SDH function is lost.

Most PGLs appear to be sporadic [46]. About 40% of all PGL cases are inherited [63] involving genes encoding four subunits (A, B, C, and D) of the SDH complex and a gene encoding SDHAF2, the SDHA-specific flavination protein [64]. Several excellent reviews of SDH loss in PGL have recently appeared [65–67]. Other causative genes for familial PGL continue to be discovered [68]. Such genes are therefore tumor suppressor genes whose mutant forms show autosomal dominant inheritance. Carriers are predisposed to PGL with tumorigenesis initiated upon random loss of heterozygosity in a susceptible cell. Because the SDH complex is essential to central metabolism and oxidizes succinate to fumarate, mutations that disrupt the complex will compromise its function. It should be pointed out that it is completely mysterious why various defects in different components of SDH structure and assembly should display different clinical phenotypes. It would be expected that defects in any SDH subunit or assembly factor would display a similar range of effects since all impact performance of the same molecular machine. Classic PGL syndromes are described below.

### 4.1. SDHA

SDHA-associated mutations are mapped to the SDHA gene on chromosome 5p15.33. Mysteriously, such mutations have very low penetrance in PGL/pheochromocytoma [69]. Thus far, six PGL patients have been reported with SDHA mutations. Five patients developed PGL and one patient had pheochromocytoma [69, 70]. SDHA mutations are also associated with Leigh syndrome [71–73]. Unlike SDH-loss PGL (a disorder displaying autosomal dominant inheritance), Leigh syndrome displays recessive inheritance requiring inherited mutations in both SDHA alleles. These patients develop early onset progressive neurodegenerative disease associated with developmental delay, lactic acidosis, ataxia, and seizures. The peculiar underrepresentation of SDHA mutations in familiar PGL is a mystery.

### 4.2. SDHB

The PGL syndrome associated with* SDHB* gene mutations was identified by Astuti and colleagues [74]; see [75, 76].* SDHB* mutations were reported to have a 77% penetrance rate by age 50 [77].* SDHB* is mapped to chromosome 1p36.1-p35. The mutation types are missense mutations (46%), frameshift mutations (23%), and splicing mutations (12%). This appears to be the most aggressive familial PGL syndrome arising from the sympathetic ganglia of the abdomen. Patients with SDHB-associated mutations are reported to be more likely to become malignant [77, 78]. Some patients with SDHB mutations have also been reported to develop renal cell carcinoma [79].

### 4.3. SDHC

*SDHC* mutations leading to head and neck PGLs were first described by Niemann and Muller [80].* SDHC* is mapped to chromosome 1q21. These PGLs tend to develop in parasympathetic ganglia with one case of an abdominal norepinephrine-secreting sympathetic paraganglioma [81]. The tumors are reported to be more often benign [82]. SDHC-associated mutations are rare and less penetrant than SDHB and SDHD mutations [46, 80, 82–84]. Reported SDHC mutations include nonsense mutations (47%), splicing mutations (33%), and large deletions (7%) [80].

### 4.4. SDHD

PGL tumors due to* SDHD* mutations were first identified by Baysal and colleagues [85]. This is the most common type of familial PGL syndrome.* SDHD* is located at the gene locus 11q23 and is maternally imprinted [77, 83]. The majority of familial PGL cases with SDHD mutations inherit the mutation from their father and will develop PGL before the age of 50 [46]. Although* SDHD*-mutant PGLs are reportedly not as aggressive as* SDHB* mutants, SDHD mutations are considered highly penetrant [77].* SDHD* mutations include frameshift mutations (40%), nonsense mutations (25%), and splicing mutations (9%) [86].* SDHD* mutations are mainly found in patients with head and neck parasympathetic PGLs occurring in multiple locations [87] and are reported to rarely metastasize [46].

### 4.5. SDHAF2

The* SDHAF2* gene is located on chromosome 11q12.1 [36, 88]. Hao and colleagues discovered that* SDHAF2* encodes a protein that inserts the FAD group into SDHA [36]. Mutation of* SDHAF2* will result in the lack of SDH activity. Thus far,* SDHAF2*-associated mutations have been linked to 15 cases of PGL [46]. In this group, the penetrance of PGL is 100% by the age of 45. The disease is confined to only the parasympathetic ganglia, and it may be maternally imprinted [89]. Since the discovery of SDHAF2, there have been multiple SDHAF-related genes that have been implicated in the assembly of the SDH complex [38, 40, 44]. It has yet to be seen whether mutations in the genes will result in PGL [68].

Assuming that PGL mutagenesis is associated with SDH pathology, it is unexplained why there are different tumor phenotypes for different kinds of loss-of-function molecular pathologies in SDH. It might be predicted that any mutation that abolishes SDH function should have the same outcome in susceptible tissues. Such mutations should, in principle, occur in SDH subunit structural genes and in genes encoding proteins that catalyze SDH maturation. It is likely that various hypomorphic alleles among the various tumor suppressor genes complicate interpretation of the genotype-phenotype correlation.

## 5. Other Tumors Associated with SDH Gene Mutations

Among additional tumors associated with SDH mutations, three autosomal dominant hereditary syndromes are of particular interest: gastrointestinal stromal tumor (GIST), GIST with PGL (Carney-Stratakis dyad), and GIST with PGL and pulmonary chondroma (Carney triad). All of these tumor syndromes are characterized by loss of SDHB in tumor immunohistochemistry staining [90–92].

GISTs are mesenchymal tumors found in the stomach and small intestine of the gastrointestinal tract [93]. The majority of GISTs are caused by KIT or platelet-derived growth factor receptor alpha (PDGFRA) mutations. About 7.5% of GISTs have mutations in the SDH genes. These tumors occur in multiple locations around the gastric wall, often metastasizing to lymph nodes [93]. It is believed that the pathogenesis of SDH-GISTs starts from germline mutations and epigenetic silencing of SDH genes. To date, SDHA mutations are most common, reported in 28% of SDH-deficient GIST [94]. SDHB, C, and D mutations together made up 20–30% of all SDH-deficient GISTs [90, 94].

The Carney-Stratakis dyad characterized by the formation of both GISTs and PGL is associated with germline mutation of* SDHB*,* SDHC*, or* SDHD* [95, 96]. The syndrome has an incomplete penetrance [97].

Finally, the Carney triad characterized by the occurrence of GISTs, PGLs, and pulmonary chondromas is an extremely rare disease. This is typically caused by mutations in KIT or PDGFAR. Thus far, SDHB, C, and D mutations have not been found in patients with Carney triad. The only link is a study showing epigenetic silencing of the* SDHC* gene locus in a Carney triad patient [98, 99]. The authors showed that the* SDHC* subunit gene was hypermethylated in some Carney triad patients. This correlated with decreased* SDHC* mRNA, loss of SDHC protein, and reduced SDH activity.

Importantly, recent integrative genomic studies have confirmed the key roles of established tumor suppressor genes in PGL but suggest that particular combinations of mutations and resulting epigenetic signatures (see below) distinguish different hereditary PGL subtypes [67].

## 6. Mechanisms of Tumorigenesis in Hereditary PGL

Two models have been proposed to explain how loss of SDH leads to PGL tumorigenesis. The first model hypothesizes that loss of SDH causes mutagenic oxidative stress with tumorigenic consequences. The second model argues that inactivation of SDH produces excess succinate, which can poison *α*-KG-dependent dioxygenases with tumorigenic consequences that are not yet understood. In this section, we will examine the evidence supporting each model.

Production of reactive oxygen species (ROS) is mapped to two main sources in mitochondria: Complexes I and III [100, 101]. Under normal conditions, Complex II is not known as a ROS producer. However, some evidence suggests that disruption of Complex II, as in SDH subunit gene mutations, can result in defective partial SDH complexes with oxidative stress, genomic instability, tumorigenesis, and decreased lifespan. The first evidence of ROS production in an SDH-deficient model came from studies of a* mev*-1 mutation in* C. elegans* [102]. The* mev*-1 gene is a homolog of the human* SDHC* gene [103]. Certain* mev*-1 missense alleles compromised the ability of SDH to interact with ubiquinone, resulting in electron leakage. The* mev*-1 mutant was hypersensitive to oxygen and had a decreased lifespan [102]. Superoxide anion and lactate levels were higher in the* mev*-1 mutant than in wild type worms [104]. Since the* mev*-1 gene is conserved in evolution, the same mutation could be studied in mouse [84] and hamster [105]. Superoxide anion levels were elevated in such animals. There was a higher frequency of DNA damage, and explanation of SDHC mutant cells into immune-compromised mice resulted in benign tumors [84]. In the hamster model, cancer phenotypes were seen in fibroblasts expressing the mutant SDHC protein [105]. Yeast models of complete SDH loss (distinguished from the* C. elegans* mev-1 missense mutation) have also confirmed increased ROS production [106–108], but no mutagenic DNA damage was detected [106]. Adding to this point, ROS production has not been observed in some recent studies of SDH-deficient models [109–112].

**Figure 4:**
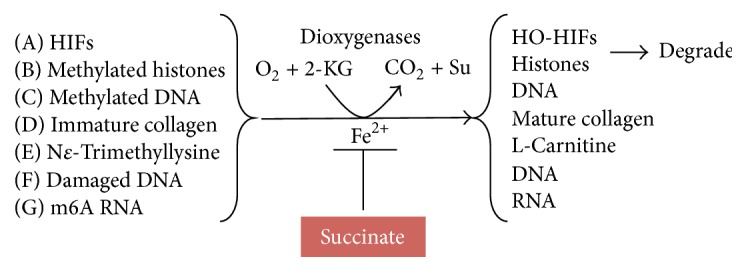
Potential *α*-KG-dependent dioxygenase targets susceptible to succinate inhibition. In SDH-deficient cells, succinate accumulates in mitochondria and diffuses into the cytosol and nucleus. Succinate inhibits *α*-KG-dependent dioxygenases resulting in accumulation of (A) HIF1*α* and HIF2*α*, (B) hypermethylation of histones, (C) hypermethylation of deoxycytidine, (D) accumulation of immature collagen, (E) accumulation of N^*ε*^-trimethyllysine, (F) damaged DNA, and (G) accumulation of N6-methyladenosine on mRNA.

The second model of PGL tumorigenesis proposes that loss of SDH function causes succinate accumulation in mitochondria. Succinate then diffuses into the cytoplasm and competitively inhibits *α*-KG-dependent dioxygenases (Figure 4) [109]. This fascinating family of iron-dependent enzymes catalyzes the splitting of molecular oxygen to modify target substrates with the simultaneous decarboxylation of *α*-KG to produce succinate as a by-product [113, 114]. Key features of this enzyme mechanism are (1) the competition between the cosubstrate *α*-KG and potential inhibitors such as succinate at the active site and (2) the importance of oxygen concentration in determining the reaction rate. The first dioxygenase proposed as a target of succinate accumulation is the prolyl hydroxylase (PHD) that modifies HIF proteins. In normoxia, HIFs are hydroxylated by PHD, which triggers HIF interaction with the von Hippel-Lindau E3 ubiquitin ligase complex. This marks HIF for proteasomal degradation. In contrast, under hypoxic conditions, the PHD reaction rate is decreased by reduction of the oxygen cosubstrate concentration and HIF is stabilized. HIF can then translocate into the nucleus and interact with the HIF1*β* subunit to activate genes that compensate for the hypoxic condition. According to this model, pseudohypoxic phenotypes are driven by loss of SDH function. It remains completely unknown how pseudohypoxia is tumorigenic and why tumorigenesis is almost completely restricted to neuroendocrine cells.

Selak and colleagues were the first to propose succinate inhibition of dioxygenases and show stabilization of HIF in an SDH loss model [109]. In their report, siRNA was used to knock down SDHD expression in HEK293 cells. This resulted in accumulation of succinate and stabilization of HIF. The authors then demonstrated that succinate could inhibit PHD activity* in vitro*, which led to the conclusion that succinate accumulation in SDH-deficient cells could inhibit PHD activity and induce HIF stabilization. Since *α*-KG and succinate compete for PHD binding, the authors showed in a separate report that increased intracellular *α*-KG levels could reverse succinate inhibition of PHD [115]. These studies did not focus on the role of oxygen concentration in these phenomena.

Since the family of *α*-KG-dependent dioxygenases includes numerous enzymes that participate in a wide range of biological processes, this model is particularly intriguing. Succinate toxicity has been extended to other dioxygenase candidate targets in PGL tumorigenesis. For example, *α*-KG-dependent Jumonji domain histone demethylases (JMHD) were shown to be susceptible to inhibition. JMHD is responsible for the removal of methyl groups from the tails of histones. Induced global histone hypermethylation could alter epigenetic control of gene expression, with potential tumorigenic consequences. In a yeast Sdh2 loss model, Smith and colleagues confirmed the absence of SDH activity [106]. Succinate accumulated about 10-fold higher in Sdh2 loss cells than in wild type cells, and JMHD activity was inhibited resulting in increased levels of histone methylation. Succinate inhibition could be overcome by increasing *α*-KG levels* in vitro*. The authors also showed that mammalian JMHD was susceptible to succinate inhibition. This work suggested for the first time that histone demethylase inhibition by succinate accumulation could alter the gene expression landscape to favor a transformed phenotype. Confirming this hypothesis, Cervera and colleagues showed that knocking down SDHB resulted in 6-fold or more dysregulation of genes that could influence proliferation, adhesion, and the hypoxia pathway [116]. More importantly, SDHB-loss cells displayed some tumor characteristics. In other work, Cervera and colleagues confirmed that loss of SDHB dysregulated histone modifications [117].

Inhibition of *α*-KG-dependent dioxygenases by succinate in SDH loss cells was subsequently extended to the TET family of DNA demethylases. TET dioxygenases remove methyl groups from 5-methylcytosine residues by first converting these residues to 5-hydroxymethylcytosine. Accumulation of 5-methylcytosine is known to correlate with repressed gene expression. Xiao and colleagues demonstrated that TET dioxygenase could be inhibited by succinate accumulation* in vitro* and in SDHA and SDHB knockdown cell culture models [118]. In this work, JMHD inhibition by succinate was also confirmed in HEK293 cells models. Cells lacking SDHA or SDHB showed accumulation of succinate and increase global 5-methylcytosine levels. Succinate inhibition of TET dioxygenase was reversible by treating the cells with a cell permeable esterified *α*-KG. In the same study, collagen synthesis was also shown to be disrupted by succinate inhibition of prolyl-4-hydroxylase, a dioxygenase that hydroxylates proline residues during collagen maturation.

These results have recently been confirmed in a study using SDHB knockout cells. Letouzé and colleagues showed that succinate accumulated in SDHB knockout chromaffin cells led to DNA hypermethylation and established a migratory phenotype [119]. Both DNA and histone hypermethylation were reportedly reversible by increasing intracellular *α*-KG levels.

The succinate accumulation hypothesis has also been supported by studies of human SDH-loss PGL specimens. Studying SDHB and SDHD mutations in families with PGL, Gimenez-Roqueplo and colleagues showed that PGL tumors accumulated succinate and the SDH complex was inactive [120]. RT-PCR measurements confirmed expression of angiogenic factors. The authors postulated that HIF activation might explain the high vascularization seen in PGL tumors. This was further confirmed by Pollard and coauthors studying SDH-related PGLs and sporadic PGLs [121]. These authors concluded that succinate could cause stabilization of HIF1*α*. In a study using biopsy material from a patient with a deleterious homozygous SDHA mutation, SDHA mutant fibroblasts showed increased HIF1*α* translocation into the nucleus [111]. In gene expression studies using micro-array and transcriptional profiling analyses, it was revealed that SDH-mutant PGLs expressed increased levels of hypoxia related genes compared to sporadic and VHL associated PGLs [122–124]. Beyond HIF stabilization, Letouzé and coauthors demonstrated that SDH-related tumors displayed a unique hypermethylation phenotype that is different from the pattern induced by RET, NF1 (neurofibromin 1), and VHL gene mutations [119]. Key genes specifying neuroendocrine differentiation were downregulated, and this might play a factor in PGL tumorigenesis. Recent work from our laboratory employed SDHB knockdown in HEK293 cells and conditional Sdhc knockout mouse embryonic fibroblasts [125]. These studies confirm succinate inhibition of PHD, JMHD, and TET enzymes but also demonstrate that these effects are exquisitely sensitive to oxygen concentration. This is in accord with the dioxygenase reaction mechanism: succinate poisoning can be overcome by increased *α*-KG and/or increased oxygen. This key observation explains hypoxia as a PGL risk factor and suggests a potential for therapeutic hyperoxia. Together, these findings suggest that succinate is an oncometabolite that can diffuse from the mitochondria to the cytosol and nucleus, perturbing a wide range of biological pathways.

## 7. Additional ***α***-KG-Dependent Dioxygenase Targets

The initial discovery that PHD, an *α*-KG-dependent dioxygenase, may play an important role in pseudohypoxic signaling in PGL inspired many groups to examine other *α*-KG-dependent dioxygenase family members in the setting of SDH loss. After PHD was shown to be inhibited by succinate [109], this effect was extended to Jumonji domain histone demethylases [106], prolyl and lysyl-hydroxylase [118], and TET dioxygenase [118, 119]. The list of *α*-KG-dependent dioxygenase targets susceptible to inhibition upon SDH loss continues to grow [126–128]. Here we discuss four potential targets (Figure 4). Each has the intriguing potential to play a role in neuroendocrine cell tumorigenesis.

ALKBH5 is a demethylase that removes the methyl group from the exocyclic amine at the 6 position of adenosine in mRNA (m6A). Similar to TET dioxygenase, the enzyme requires *α*-KG and molecular oxygen generating carbon dioxide and succinate as by-products. Since its substrate requirement is identical to TET dioxygenase, we predict that ALKBH5 will be inhibited by succinate in SDH loss cells. 6-Methyladenine (m6A) is a newly recognized mRNA mark linked to a growing number of functions. In mammalian cells, m6A markers are thought to be important for RNA binding by proteins involved in mRNA export (from the nucleus to the cytosol), RNA metabolism, and mRNA processing [129]. Consistent with ALKBH5 as a dioxygenase that reverses this RNA methylation, depletion of ALKBH5 has been shown to cause accumulation of m6A in mRNA and to be associated with male infertility [129]. m6A in mRNA can also play a role in RNA degradation. For instance, YTHDF2 protein has been shown to bind to m6A in some mRNA positions and relocate RNA to sites of degradation [130]. Thus, it is possible that succinate poisoning of ALKBH5 leads to accumulation of m6A with tumorigenic consequences related to mRNA metabolism.

Another *α*-KG-dependent dioxygenase family predicted to be inhibited by succinate accumulation is the hABH dioxygenases. These DNA repair proteins remove potentially mutagenic alkylation damage from DNA. These enzymes bind to the methylated base, flip it out from the normal stacked DNA structure while temporarily inserting a hydrophobic amino acid at the repair site [131], and then catalyze oxidative demethylation of the base. Inhibition of hABHs is predicted to result in increased DNA methylation with potentially mutagenic consequences.

Biosynthesis of carnitine involves two *α*-KG-dependent dioxygenases: 6-N-trimethyllysine-3-hydroxylase (first enzymatic step) and *γ*-butyrobetaine hydroxylase (final enzymatic step) [128]. L-Carnitine is required to transport fatty acids from the intermembrane space into the matrix for fatty acid metabolism by *β*-oxidation pathway. Succinate accumulation therefore has the potential to inhibit carnitine biosynthesis and alter fat metabolism.

## 8. Relationship between Succinate Accumulation and Accumulation of Other Dioxygenase Inhibitory Metabolites in Cancers

Parallel to the succinate accumulation hypothesis, defects in three other TCA cycle enzymes have been linked to competitive inhibition of *α*-KG-dependent dioxygenases and tumor formation: isocitrate dehydrogenase (IDH), fumarate hydratase (FH), and malate dehydrogenase 2 (MDH2). This section will focus on the defects and the mechanisms that link these enzymes to cancers.

There are three IDH isoforms (IDH1, IDH2, and IDH3). IDH1 is localized to the cytosol and peroxisomes [132]. IDH2 is present in both cytosol and mitochondria, whereas IDH3 is only found in the mitochondria. In the TCA cycle, IDH catalyzes the decarboxylation of isocitrate to *α*-KG using NADP+ as a cofactor and producing NADPH and CO_2_ in the process.

**Figure 5:**
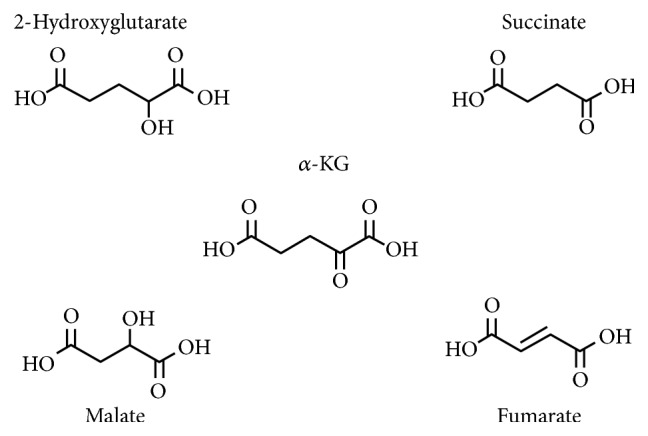
Structural similarity between TCA cycle oncometabolites. IDH gain of function mutations produces 2-hydroxy-*α*-KG. SDH and FH mutations lead to accumulation of succinate and fumarate, respectively. MDH2 mutations result in increase of malate and fumarate.

Unlike SDH mutations, somatic mutations of IDH result in a gain of function [133, 134]. The majority of these mutations involved amino acid substitution at IDH1 codon 132 (~92% are missense mutations resulting in the R132H substitution). Remarkably, this change alters the enzyme chemistry from a dehydrogenase to a reductase that reduces *α*-KG to 2-hydroxyglutarate [135]. This leads to depletion of *α*-KG and accumulation of 2-hydroxyglutarate. These compounds are structurally similar (Figure 5), suggesting that 2-hydroxyglutarate competes unproductively with *α*-KG for the active site of *α*-KG-dependent dioxygenases. Indeed, this has been confirmed. In cells with IDH R132H mutations, there is hypermethylation of histones and DNA [136, 137]. Interestingly, even though these metabolites presumably affect the same dioxygenases, mutations in IDH1/2 are linked to glioma and acute myeloid leukemia [138], but not to PGL/pheochromocytoma. Equally mysterious is the fact that no SDH mutations have been found in glioma and practically no IDH mutations have been found in PGL.

FH is a homotetrameric protein in the TCA cycle that catalyzes the hydration of the double bond of fumarate to generate malate [1]. Like SDH, FH is a tumor suppressor, and loss of heterozygosity will predispose FH mutant carriers to develop disorders including renal cell cancer, cutaneous and uterine leiomyomas, and encephalopathies [139]. Interestingly, a subset of patients with FH mutations develop PGL and pheochromocytoma [140]. This could be explained by a shared oncometabolite mechanism of tumorigenesis between SDH-related PGLs and FH-deficient PGLs. It has been reported that FH deficiency results in accumulation of fumarate. Because fumarate has a higher affinity for *α*-KG-dependent dioxygenases than succinate, it is expected to be a global inhibitor (Figure 5) [141–143]. To date, FH deficiency has been shown to stabilize HIF1*α* [144] and cause accumulation of histone and DNA methylation [118].

The third TCA cycle enzyme that is linked to inappropriate metabolite accumulation is MDH2 [68]. MDH2 is a mitochondrial enzyme that catalyzes the oxidation of malate to oxaloacetate. There are two isoforms of MDH: MDH1 and MDH2 [1]. MDH1 is found exclusively in the cytosol and is the primary enzyme in the malate-aspartate shuttle. It has been reported that MDH2 mutation can result in 2-fold increase of malate and fumarate levels (Figure 5) [68]. More importantly, MDH2 mutation has recently been linked to PGL. Currently, the mechanism of tumorigenesis is unknown, but there is an obvious and suspicious similarity to the mechanism of SDH and FH mutations.

Together, defects in TCA cycle enzymes with accumulation of dicarboxylic acid oncometabolite variants highlight the importance of continuing research in this field. It is puzzling and mysterious that these defects trigger a common pseudohypoxia pathway and aberrant epigenetic landscape yet have such distinctly different pathological outcomes. Questions regarding tissue specificity and oncogenic pathways will need to be answered in future research. Given the general potential of dicarboxylic acids to inhibit dioxygenases, it seems likely that additional metabolic enzymes will be defined as tumor suppressors.

## 9. Conclusion

The field of oncometabolite-driven tumorigenesis has gained new interest with discoveries of germline mutations (SDH, isocitrate dehydrogenase, fumarate hydratase, and malate dehydrogenase) linking the TCA cycle with tumorigenesis. It is hypothesized that these mutations result in accumulation of metabolites that competitively inhibit *α*-KG-dependent dioxygenases. Because there are many enzymes in this family, their inhibition could have profound implications in a wide range of cell regulatory pathways including hypoxic response, collagen maturation, and epigenetic control (histone, DNA, and RNA methylation) of gene expression. Thus, the succinate accumulation hypothesis for familial PGL has been inspirational in proposing part of a mechanism for loss of SDH function leading to tumorigenesis. Though the actual linkage to tumorigenesis remains unknown, this focus places emphasis on the peculiar mechanism of *α*-KG-dependent dioxygenases and potential approaches to reverse succinate inhibition. In particular, the enzyme reaction mechanism is predicted to be highly sensitive to the balance between succinate and *α*-KG, encouraging the concept of nontoxic *α*-KG analog therapy. Likewise, the reaction mechanism is profoundly sensitive to oxygen concentration, a concept not emphasized in work to date. Our recent experiments [125] reexamined the succinate accumulation hypothesis under different oxygen conditions in new SDH-deficient mammalian cell models. We showed that *α*-KG-dependent dioxygenases are synergistically inhibited by succinate and hypoxia, as predicted from first principles of the enzyme reaction mechanism. Finally, the tissue specificity of tumorigenesis secondary to SDH loss remains a puzzle. We suggest that perhaps neuroendocrine tissue is predisposed to respond uniquely to chronic pseudohypoxia when HIF is perpetually active. Perhaps this chronic signal in neuroendocrine tissue eventually triggers mitogenesis in a failed homeostatic mechanism wherein the tissue expands fruitlessly in an attempt to increase catecholamine section to induce oxygenation.
